# Meta-device for sensing subwavelength lateral displacement

**DOI:** 10.1038/s41377-025-02067-7

**Published:** 2026-01-12

**Authors:** Shufan Chen, Yubin Fan, Hao Li, Xiaodong Qiu, Ben Wang, Lijian Zhang, Shumin Xiao, Din Ping Tsai

**Affiliations:** 1 Department of Electrical Engineering and State Key Laboratory of Optical Quantum Materials, City University of Hong Kong, Kowloon, Hong Kong SAR, 999077 China; 2 Ministry of Industry and Information Technology Key Lab of Micro-Nano Optoelectronic Information System, Guangdong Provincial Key Laboratory of Semiconductor Optoelectronic Materials and Intelligent Photonic Systems, Harbin Institute of Technology, Shenzhen, China; 3 National Laboratory of Solid State Microstructures, Collaborative Innovation Center of Advanced Microstructures, College of Engineering and Applied Sciences, Jiangsu Physical Science Research Center, Nanjing University, Nanjing, 210093 China; 4 State Key Laboratory of Terahertz and Millimeter Waves, City University of Hong Kong, Kowloon, Hong Kong SAR, 999077 China; 5 Department of Physics, City University of Hong Kong, Kowloon, Hong Kong SAR, 999077 China

**Keywords:** Metamaterials, Imaging and sensing

## Abstract

Accurate transverse displacement measurement is essential for precise mask-to-wafer positioning in lithography. While lateral displacement metrology has achieved nanometer-level precision, the limitations imposed by coherent state and grating challenge in-situ measurement speed and precision. Here, we introduce a two-photon state transverse displacement measurement method utilizing a polarization gradient metasurface by employing two-photon state interference. Compared with the classical method, our new method can experimentally reduce the number of detected photons to around 3% with equivalent precision. These attributes make the two-photon state polarization gradient metasurface approach highly suitable for integration with semiconductor lithography processes and show its promise in realizing equivalent measurement precision within notably shorter acquisition durations, providing a robust solution for next-generation transverse displacement measurement requirements.

## Abstract (web summary)

Two-photon-enhanced lateral displacement metrology with polarization gradient (geometric phase) metasurfaces achieves 97% photon number reduction for high-speed nanoscale mask-wafer alignment in next-generation lithography.

## Graphical abstract

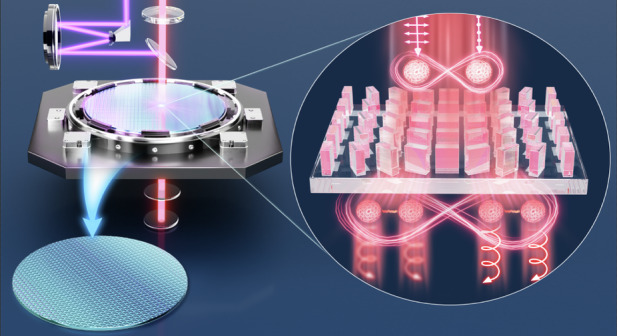

## Introduction

Precise displacement measurement using optical methods has emerged as a fundamental cornerstone across diverse scientific and technological domains, including gravitational wave detection, super-resolution microscopy, and semiconductor manufacturing lithography processes. Lithography constitutes the fundamental pillar of the modern semiconductor industry, playing a crucial role in enabling the intricate design and mass production of integrated circuits at the micro- and nanoscale. In the lithography industry, alignment methods are categorized into overlay and alignment, each serving different purposes. Alignment involves adjusting the mask’s position before exposure to ensure its marks align with those on the wafer, while overlay assesses positional deviations between layers post-exposure. Very recently, alignment methods using Moiré fringes^1^ and high-order differentials^2^ have also been proposed, enriching the toolbox of alignment methods. Optically precise displacement measurement facilitates the production of semiconductor devices with sub-20 nm resolution through multiple patterning^3^. Accurate measurement of transverse displacement is crucial for properly positioning the photomask relative to the wafer^4^.

The demand for higher precision in lithography has catalyzed the development of displacement measurement methodologies. Structured light has emerged as a powerful tool in displacement measurement, offering innovative approaches through spatially varying distributions of amplitude, phase, polarization, and orbital angular momentum states^5–9^. Concurrent advancements in nanophotonics have significantly improved the precision of displacement measurements, reducing alignment requirements for structured light as well as the size of measurement components^10–13^. As a significant part of nanophotonics, metasurfaces have brought a transformative era in optical metrology, like refractive index measurement^14^, phase singularity optical ruler^15^, far-field holographic patterns^16^, and polarization gradient metasurface^17–20^, providing unparalleled control over light manipulation at the nanoscale^21–29^. These artificially engineered planar structures allow for precisely shaping optical wavefronts and polarization states. By combining novel physical concepts and methods, such as bound states in the continuum and matchless nanocavities, metasurfaces show their potential as a platform for physics research, like strongly enhanced 2D materials multi-photon nonlinear process^30^. These artificially engineered planar structures allow for precise field manipulation, shaping optical wavefronts and polarization states. Metasurfaces present compelling advantages for precision displacement measurement in lithography, particularly due to their inherent compatibility with established semiconductor manufacturing processes. This unique compatibility serves as a critical bridge between optical technologies and existing fabrication infrastructure. Furthermore, achieving comparable precision with reduced photon detection under constant light source intensity enables less time measurement cost, meeting a crucial requirement in the lithography industry applications.

However, while these achievements have realized highly compatible, high-precision position and movement measurement, the wave-like nature of coherent state imposes constraints on further on-chip and in-situ measurement speed and precision. Existing approaches necessitate the detection of a substantial number of photons to achieve high precision. The extensive duration required to detect this quantity of photons poses a significant limitation on the ability of coherent state-based methods to reduce measurement time further. Metasurface for multi-photon state manipulation has witnessed tremendous advancements in recent years^31–33^, including polarization entangled Bell states generation^34^, quantum CZ gate realization^35^, multiprotocol quantum key distributions^36^, vectorial meta-holography^37^, and multimodal imaging^38^. Based on the above works, utilizing two-photon state effects at the micro and nanoscale holds promise for addressing the issues and enables the attainment of high precision while utilizing a reduced number of photons, thereby effectively decreasing the overall measurement duration.

In this work, we present a cutting-edge technique for transverse displacement measurement utilizing a polarization gradient metasurface, which is compatible with contemporary lithography methods. By employing two-photon state interference, our method demonstrates significant potential for enhancing resolution and experimentally demonstrates that equivalent precision positioning can be sustained while utilizing approximately only 3% of the photon count required by classical methods. Furthermore, our system can measure velocity within a speed range of 20–5000 nm/s. These attributes make our displacement measurement technique highly suitable for integration with semiconductor lithography processes, showing its promise in realizing equivalent measurement precision, notably reducing the number of detecting photons, and providing a robust solution for the measurement requirements of next-generation transverse displacement measurement technologies.

## Results

### Working principle of the two-photon state in transverse displacement measurement

**Fig. 1:**
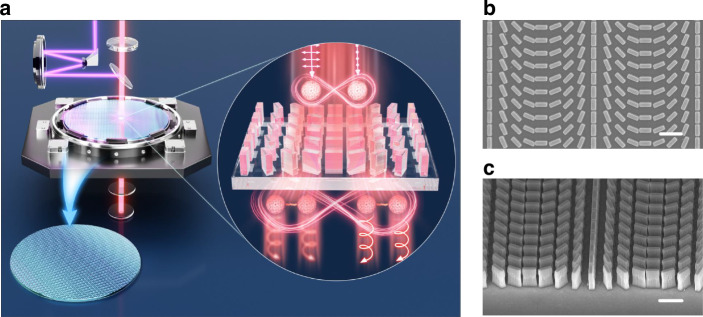
Metasurface for sensing lateral displacement. **a** Schematic of polarization gradient metasurface precision process control. Main: The diagram illustrates that the purple light signifies ultraviolet light used for exposure, while the red light denotes the measurement light of two photons. Inset: two photons with orthogonal polarization are converted into entangled photons upon passing through the metasurface, resulting in both photons being emitted at the same angle. **b** Top and **c** tilt of the SEM image. Scale bar: 1 μm

The schematic of our work is shown in Fig. 1a. The metasurface we have designed is a polarization gradient metasurface, functioning as a polarization beam splitter for right circularly polarized photons (denoted as *|R*〉) and left circularly polarized photons (denoted as *|L*〉) based on geometric phase, also known as the Pancharatnam–Berry (PB) phase. The polarization gradient metasurface is fabricated based on a 960 nm TiO_2_ film (Supplementary Material, Section 1). Following the careful design of the unit cell (Supplementary Material, Section 2), we proceeded with the fabrication of the sample using a standard top-down semiconductor fabrication process (Supplementary Material, Section 3). Top and tilt scanning electron microscope (SEM) images of the sample are shown in Fig. 1b, c.

**Fig. 2:**
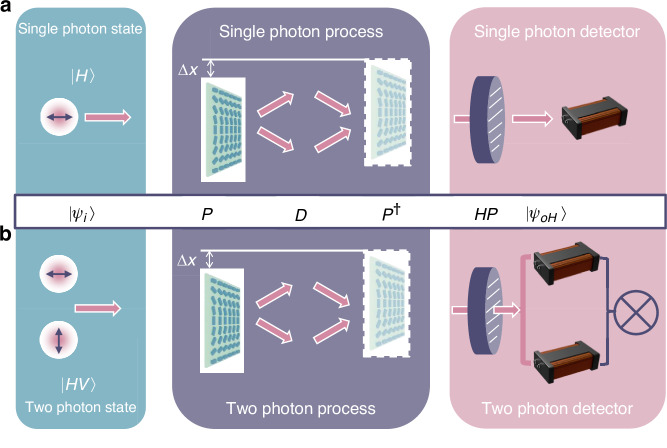
Working principle of phase gradient (PB phase) metasurface. Schematic diagram of **a** single-photon interference and **b** two-photon interference

Polarization gradient metasurfaces are used as circular polarization beam splitters to help detect displacement shifts^18^ and create two-photon states^31^. These processes can be clearly explained using the idea of linear systems, where each step is seen as a specific action. First, an incoming light state *|ψ*_*i*_〉 in {*|H*〉, *|V*〉} basis, undergoes a basis change *P*, which means its reference basis is adjusted to {*|R*〉, *|L*〉} basis. Next, a shift or displacement *D* is added from the movement of the polarization gradient metasurface to this state. After this, the state is changed back to its original basis via *P*^†^, returning it to the starting basis. The final step involves polarization measurement using a linear horizontal polarizer *HP*, which helps us understand the new state of the light *|ψ*_*oH*_〉 under *|H*〉 basis. These steps together provide a way to convert displacements into measurements of changes in two-photon states. The complete expression is,

1 $$|{\psi }_{{oH}}{{\rangle }}={HP}{P}^{\dagger }\,D\,P|{\psi }_{i}{{\rangle }}$$

where$$\left|{\psi }_{i}\right\rangle =\left(\begin{array}{c}H\\ V\end{array}\right),{P}=\frac{1}{\sqrt{2}}\left(\begin{array}{cc}1 & 1\\ i & -i\end{array}\right),\,D=\left(\begin{array}{cc}0 & {e}^{i\,4\varphi }\\ {e}^{-{i}\,4\varphi } & 0\end{array}\right),{HP}=\left(\begin{array}{cc}1 & 0\\ 0 & 0\end{array}\right),{P}^{\dagger }$$ is the Hermitian conjugation of *P*. $\Delta \varphi =\frac{2\pi }{\Lambda }\Delta x$ is the phase change induced by displacement $\Delta x$, Λ is the period of polarization gradient metasurface. We can easily get the outcomes of the projection measurement of a coherent state or single photon, taking the incident state as *|H*〉, detection performed after a horizontally polarized beam splitter, $\left\langle H|{\psi }_{oH}\right\rangle ={\cos} 2{\varphi}$. The schematic diagram is shown in Fig. 2a. The same process can be easily discussed on a two-photon basis regarding two-photon cases. Especially when the incident state is *|HV*〉, after a horizontally polarized beam splitter, detection performed on *|HH*〉 basis, the final photon counting rate follows $\left\langle {HH}|{\psi }_{{oH}}\right\rangle =-\frac{1}{2}\sin 4\varphi$, and the schematic diagram is shown in Fig. 2b. See Supplementary Material, Section 4 for more details. Compared to the case of a coherent state or single-photon state as a light source, the variation of *φ* is amplified by a factor of 2, which shows the intuitive principle of measuring displacement and the distinction between coherent states and two-photon states.

The utilization of the two-photon states offers significant advantages over the coherent state. These advantages arise from the fact that the Quantum Fisher information (QFI) associated with the two-photon state is twice that of the QFI for a single-photon or coherent state with the same photon count. Specifically, for measurement iterations denoted as *n*, the precision of this method, described in terms of Fisher information (FI), increases proportionally to *n*. This contrasts with conventional measurement techniques employing coherent light, where the precision only improves proportionally to $\sqrt{n}$. Our method demonstrates a clear superiority in enhancing measurement precision, particularly when the number of measurements *n* is substantial. According to the Cramér-Rao Bound, the lower bound for the variance of the estimated phase is defined as ${\left(\Delta \varphi \right)}^{2}\ge \frac{1}{{nQFI}}$. The phase can be represented in terms of displacement as the following the relationship $\Delta \varphi =\frac{2\pi }{\varLambda }\Delta x$. Consequently, the lower bound for the variance of the relative displacement is given by ${\left(\Delta x\right)}^{2}\ge \frac{1}{{nQFI}}\frac{1}{{\left(\frac{2\pi }{\varLambda }\right)}^{2}}$.

**Fig. 3:**
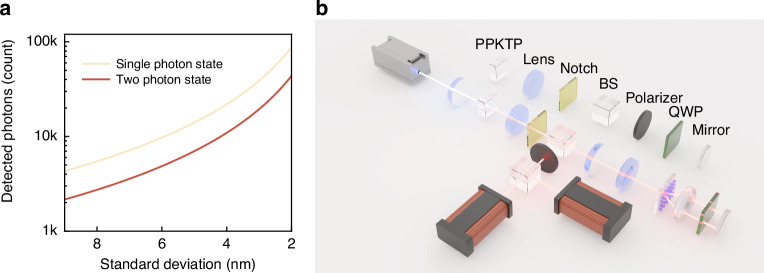
Measurement improvement through metasurface combined with two-photon states. **a** Comparison of measurement advantages between the single-photon state and the two-photon states. The two-photon states require fewer detected photons than single-photon states to achieve the same standard deviation. **b** Experimental setup of polarization gradient metasurface transverse displacement measurement by two-photon interference

The ${QFIs}$ calculated are ${QFI}=4$ for the two-photon states, with a photon number $N=2$, and ${QFI}=1$ for the single-photon state, with a photon number $N=1$. Let $\varLambda =5000{\rm{nm}}$. As illustrated in Fig. 3a, when compared to twice the single-photon states, it is evident that for the same standard deviation, the number of detected photons $N$ for the two-photon states is 50% of that for twice the single-photon state. The coincidence counting process significantly mitigates the noise caused by ambient light and effectively filters out photons lacking phase information. These intrinsic properties of the two-photon states and measurement processes underscore their potential to enhance measurement precision beyond classical limits and reduce the number of detected photons while achieving the same measurement precision.

The above two principles, namely, the description of two-photon states displacement shifts and ${QFI}$, ensure our declared advantages, specifically reducing the number of photons required for displacement measurement. Our principles provide a theoretical guidance for transverse displacement measurements involving two-photon states for researchers, including semiconductor lithography production^39^ and metasurface applications. Compared to classical methods, our method achieves higher precision while requiring two orders of magnitude fewer photons. In the following, experimental results are presented to demonstrate how this principle reduces the number of detected photons while achieving the same standard deviation.

Our experimental setup is depicted in Fig. 3b. We utilized collinear Type-II parametric down-conversion to generate degenerate photon pairs at 810 nm. The spatial mode of the light source was carefully adjusted using a combination of multiple lenses, which were elaborately designed to illuminate the polarization gradient metasurface appropriately. This design enabled us to achieve high fiber coupling efficiency. Photon counting was performed using single-photon counting modules. See Methods and Supplementary Material Section 4 for more details. We made samples that exhibit a phase shift of 2π over a distance of 5 µm, 7.5 µm, and 10 µm (Supplementary Material, Section 5) to show the compatibility with contemporary lithography methods.

### Transverse displacement sensing

**Fig. 4:**
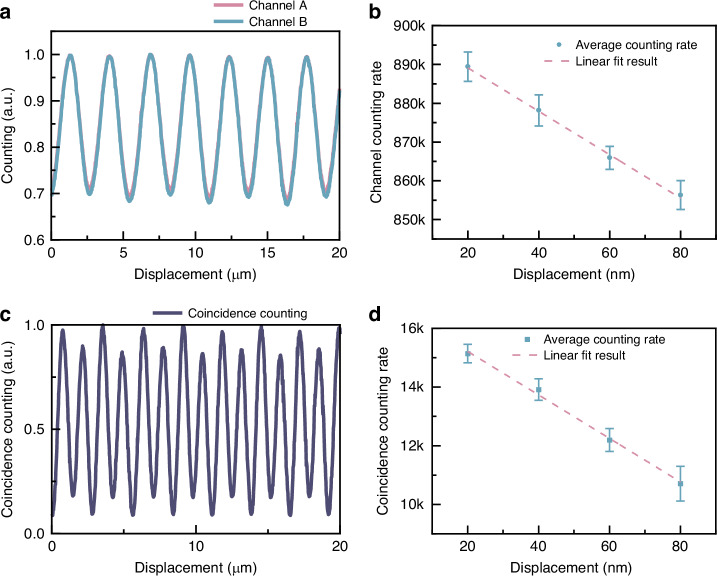
Static results of polarization gradient metasurface transverse displacement measurement. **a** Straight line and **b** incremental measurement results of single photon counting (one channel in coincidence measurement). **c** Straight line and **d** incremental measurement results of coincidence counting

To verify the system’s positioning properties, we first verified the experimental setup (Supplementary Material, Section 6) and then selected a polarization gradient metasurface with 5 µm and conducted a straight-line movement of the translation stage. The detailed interference scheme of the wavefronts is shown in Supplementary Material, Section 7. To validate the system’s measurement capabilities, we first implemented minor incremental displacements to the polarization gradient metasurface via the piezoelectric stage. We observed sinusoidal signal variations corresponding to these displacements, as depicted in Fig. 4a. This allowed us to effectively gauge the system’s characterization by analyzing the resultant changes in the observed signals, thereby providing a reliable measure of performance for two-photon state measurements.

In order to achieve precise calibration in the classical scenario, measurements were conducted within the domain where displacement changes exhibited an almost linear response, particularly near the sine wave’s most inclined section. Figure 4b illustrates each data point as the result of 50 repeated measurements. With the stage moving in 20 nm increments, each measurement point was clearly distinguishable. The standard deviations for these measurements were 6.73, 7.19, 5.31, and 6.67 nm, respectively, thereby showing the system’s proficiency in attaining nanometer-level precision in the classical case.

The measurement results are shown in Fig. 4c, d. During the movement of the piezoelectric stage, we observed that the two-photon coincidence count intensities exhibited a sinusoidal variation, doubling the frequency of the single-photon signal, as Fig. 4c shows. This result indicates that the frequency of the sinusoidal oscillations is indeed doubled compared to classical conditions, as evidenced by single-channel counts. We can reach the minimum points of the sine wave and realize visibility up to 84%. The contrast reduction is due to the error caused by accidental coincidence counting. As illustrated in Fig. 4d, each measurement point was also clearly discernible when the stage was moved in 20 nm increments. The standard deviations for the measured points were 4.28, 4.98, 5.28, and 7.95 nm, respectively, highlighting the system’s ability to achieve nanometer-level precision. Our experimental results are highly relevant for the prevalent 65 nm process, which is widely used in mobile, computing, automotive, Internet of Things, and wearable tech. This validates our research and could drive progress in related industries.

The findings presented herein substantiate the premise that two-photon interference facilitates enhanced measurement precision surpassing that of classical methods. Notably, the two-photon approach achieves a reduced measurement standard deviation, even when the photon count is significantly decreased to merely 3.4%, 3.2%, 2.8%, and 2.6% relative to the photon numbers typically required by classical methodologies, comparing Fig. 4b with d. This denotes an improvement approaching two orders of magnitude in the photon quantity, with only around 3% of the detected photon number needed to achieve comparable measurement precision. More details on error analysis can be found in Supplementary Material, Section 8. The coincidence counting process significantly mitigates the noise caused by ambient light and effectively filters out photons lacking phase information. The measurement standard deviation can decrease by a factor of 0.707 when the photon numbers detected in two-photon and single-photon measurements are equal. The results show that the standard deviations for both types of measurements are consistent. Ultimately, the advantage of a two-photon state is its ability to carry more information than a single-photon state, leading to comparable standard deviations with fewer photons.

### Calibration of the dynamic range

**Fig. 5:**
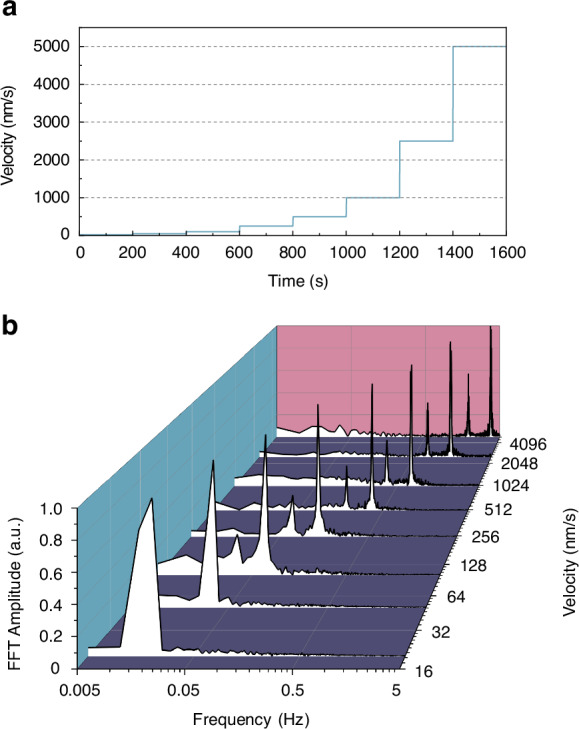
Calibration of the dynamic range of the measurement. **a** The moving speed of the piezoelectric translation stage over time, and **b** the corresponding measurement results

In semiconductor lithography involving multiple exposures, the movement of the measured object often compromises measurement precision. It is critical to evaluate how this movement impacts the precision of such measurements. To improve frequency accuracy, we capture more measurement cycles at lower speeds within a constant sampling time period. This was accomplished using a piezoelectric displacement stage moving at a fixed velocity, as depicted in Fig. 5a, to calibrate the dynamic range. Our results demonstrate that by maintaining a constant speed for the translation stage and applying Fast Fourier Transform (FFT) analysis, the system’s dynamic range was effectively calibrated, as shown in Fig. 5b. The system performed reliably at speeds ranging from 20 to 5000 nm/s, with measured peaks aligning with expected FFT outcomes. Minor peaks at half the main frequency were identified as oscillations due to accidental coincidences. This system’s enhancement of frequency accuracy at low speeds supports precise control in complex multiple-exposure processes. By assessing the influence of object movement on measurement precision, the multiple-exposure process can be optimized, thereby improving the reliability of the measurement results.

In lithography, a coarse stage handles large-scale movement, while a fine stage allows for precise micro-level adjustments^40^. For the fine stage, velocity feeding is set at 100 nm/s for nanometer-level precision^41^. Our dynamic range of moving speed meets the requirements of such a fine-stage system.

## Discussion

In this work, we have developed a transverse displacement measurement device using a polarization gradient metasurface that is compatible with semiconductor processes. By leveraging polarization gradient metasurface and two-photon state interference, the proposed method enhances displacement measurement and alignment in lithography by achieving nanometer-scale precision ideal for the pre-alignment and alignment process, using a low-photon-count process to minimize exposure interference. It maintains precision with only about 3% of the detected photons. Additionally, the system is applicable in various fields, such as precision machining and vibration compensation, suggesting broader future applications. The system performed reliably at speeds ranging from 20 to 5000 nm/s, meeting the requirements of lithography processes in semiconductor manufacturing. This allows for the measurement of rapidly moving chips, meeting the manufacturing demands of lithography systems. A short introduction to modern photolithography alignment technology can be found in the Supplementary Material, Section 9. The potential impact of our approach is significant: with the number of photons with the same generation rate from the light source, the integration time is reduced by two orders of magnitude. Our method potentially enables the semiconductor industry to save 97% of time costs during the lithography exposure process.

Several strategies can be employed to achieve further improvement in resolution and precision. One approach involves shortening the interferometer arm length through meticulously designed integrated optical paths, which minimize jitters. Additionally, utilizing multi-photon states with higher photon numbers can lead to higher precision while collecting the same number of photons. Currently, employing $N=2$ results in a precision of $\frac{1}{\sqrt{2}}$; however, using multi-photon states with $N$ photons can achieve a precision of $\frac{1}{\sqrt{N}}$. Moreover, enhancing the indistinguishability of SPDC photon pairs by narrowing the bandwidth of bandpass filters can improve interference contrast. While this method increases indistinguishability, it may also decrease the efficiency of the optical path, thus requiring a careful balance to be maintained. Reducing the operating wavelength represents an effective approach for enhancing resolution, as Supplementary Material, Section 10, but this method is constrained by the working wavelength of the crystal used in generating two-photon states.

Our method provides a robust, non-intrusive, and highly accurate approach to measuring transverse displacement, addressing critical challenges in advancing lithography technology and promoting the application of metasurfaces.

## Methods

### Fabrication

The fabrication process of the TiO_2_ metasurface involves several steps. First, a 960 nm TiO_2_ film is deposited onto an ITO glass substrate at 0.6 Å/s. An 80 nm PMMA film is then spin-coated and baked at 180 °C for 1 h. The PMMA resist is exposed to an electron beam (30 kV) and developed in a MIBK/IPA solution at 0 °C for 30 s to form nanostructures. A 30 nm Cr film is applied as a hard mask using an electron beam evaporator at 0.3 Å/s, followed by a lift-off process in the PG remover solution. Reactive ion etching is used to etch the TiO_2_ layers, and finally, the residual Cr film is removed by immersing the sample in a chromium etchant for 10 min. See Supplementary Material, Section 3 for detailed information.

### Experimental setup

The experimental setup involves generating orthogonally polarized biphotons through the process of SPDC. Initially, a continuous wave single-longitudinal-mode laser operating at 405 nm is focused onto a PPKTP crystal using a lens with a focal length of 20 cm. The pump light at 405 nm is subsequently filtered out using a narrow-bandpass filter. The SPDC photons are first transmitted through a 50:50 beam splitter and then enter a 4f system designed to relay the Gaussian beam, thereby ensuring that the waist position of the SPDC beam coincides with the location of the polarization gradient metasurface. Upon passing through the polarization gradient metasurface, incident orthogonal linearly polarized photons are split into two beams with left-handed and right-handed circular polarizations. A lens with a focal length of 5 cm, positioned behind the polarization gradient metasurface, is used to deflect the beams such that they can normally incident onto a quarter-wave plate and a mirror. The quarter-wave plate is utilized to impart opposite circular polarization directions to the photons upon their first and second incidences on the polarization gradient metasurface. Consequently, the returning beams are deflected by the same angle and retrace their original paths. The reflected light from the 50:50 beam splitter is then projected onto a horizontal polarization state using a polarizing beam splitter. Finally, the biphotons are directed through a fiber beam splitter to achieve projection measurements. The coincidence counting method represents a linear detection technique, which is distinct from two-photon absorption, as shown in Supplementary Material, Section 11. This setup ensures precise control and measurement of the polarization states of the biphotons, facilitating detailed analysis of their states’ properties.

To improve the generation rate of two-photon states, we can adopt more efficient light sources. For example, there are photon pairs with a pair generation rate of 41.77 GHz mW^−1^, as reported^42^, and a photon-pair source at cryogenic conditions shows a high brightness of 8 GHz mW^−1^, as described^43^. These more efficient sources can potentially address the issue of the initial photon budget and further enhance the performance of our two-photon-based measurement approach.

### Velocity measurement

Speed measurement necessitates a relatively high sampling rate, making it impractical to compute the coincidence count concurrently with sampling. Consequently, we employed the strategy of recording photon arrival times first and subsequently calculating the coincidence count. This approach is particularly crucial for measurements at speeds exceeding 2500 nm/s. At higher speeds, the system’s limitation is a low coincidence count rate, leading to significant spectral broadening after the FFT. Although the FFT peak can still be identified, its position is inaccurate. Conversely, at lower speeds, the system’s limitation is the inability to determine the complete cycle within a given time frame, resulting in spectral leakage and an indeterminate peak position. The double oscillation frequency introduced by super-resolution offers a solution to the time-constrained challenges of low-speed measurements.

## Supplementary information

Supplementary Information

## Data Availability

The primary data supporting the findings of this study are available within the Article and its Supplementary Information. All data generated in the present study are available from the corresponding author upon reasonable request.

## Code availability

All simulation and data processing codes in the present study are available from the corresponding author upon reasonable request.
