# Supplementary material for: Meta-device for sensing subwavelength lateral displacement
Source: Light Sci Appl. 2026 Jan 12;15:68. doi: 10.1038/s41377-025-02067-7 (PMC12791143; doi:10.1038/s41377-025-02067-7)
Supplement: Supplementary file 1 — Supplementary Information [file 41377_2025_2067_MOESM1_ESM.docx]

Supplementary Information for

**Meta-device for Sensing Subwavelength Lateral Displacement**

Shufan Chen^1,†^, Yubin Fan^1,†,*^, Hao Li^2,†^, Xiaodong Qiu^1^, Ben Wang^3^, Lijian Zhang^3,*^, Shumin Xiao^2,*^ and Din Ping Tsai^1,4,5,*^

*1 Department of Electrical Engineering, City University of Hong Kong, Kowloon, Hong Kong SAR, China.*

*2 Ministry of Industry and Information Technology Key Lab of Micro-Nano Optoelectronic Information System, Guangdong Provincial Key Laboratory of Semiconductor Optoelectronic Materials and Intelligent Photonic Systems, Harbin Institute of Technology, Shenzhen, China.*

*3 National Laboratory of Solid State Microstructures, Collaborative Innovation Center of Advanced Microstructures, College of Engineering and Applied Sciences, Jiangsu Physical Science Research Center, Nanjing University, Nanjing, 210093, China.*

*4 State Key Laboratory of Terahertz and Millimeter Waves, City University of Hong Kong, Kowloon, Hong Kong SAR, China.*

*5 Department of Physics, City University of Hong Kong, Kowloon, Hong Kong SAR, China.*

**^*^**Corresponding Authors**,** E-mails:

yubinfan@cityu.edu.hk; lijian.zhang@nju.edu.cn; shumin.xiao@hit.edu.cn; dptsai@cityu.edu.hk

^†^These authors contributed equally to this work.

Contents

[Supplementary Note 1: Refractive index of TiO_2_ film 3](#_Toc200118047)

[Supplementary Note 2: Optimization process of unit cell size 4](#_Toc200118048)

[Supplementary Note 3: Fabrication process 5](#_Toc200118049)

[Supplementary Note 4: Selecting the appropriate type of crystal and measurement basis 6](#_Toc200118050)

[Supplementary Note 5: Efficiency and divergence angle of the sample 9](#_Toc200118051)

[Supplementary Note 6: Two-photon behavior in experiments 12](#_Toc200118052)

[Supplementary Note 7: Detailed interference scheme of the wavefronts 13](#_Toc200118053)

[Supplementary Note 8: Error analysis 14](#_Toc200118054)

[Supplementary Note 9: Modern photolithography alignment technology 16](#_Toc200118055)

[Supplementary Note 10: Effect of changing the working wavelength of the metasurface 17](#_Toc200118056)

[Supplementary Note 11: Working principle of coincidence counting 18](#_Toc200118057)

# Supplementary Note 1: Refractive index of TiO_2_ film

**Fig. S1:** The refractive index of TiO_2_ film

To characterize the refractive index, we employed an ellipsometer to measure both the thickness and refractive index of the sample. The Refractive index n and extinction coefficient k are illustrated in Fig. S1. We fabricate the Metasurface based on this substrate.

# Supplementary Note 2: Optimization process of unit cell size


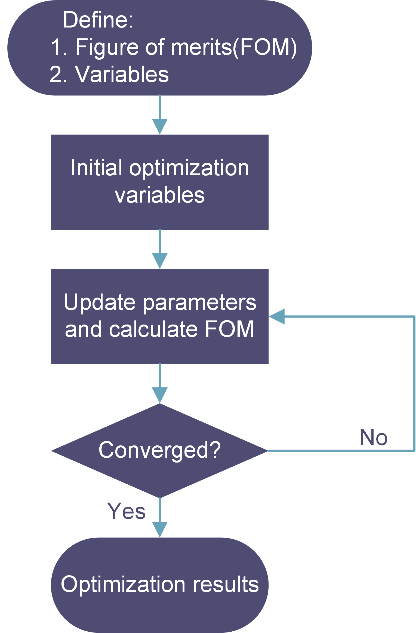


**Fig. S2:** Flow chart of unit cell size process

To achieve the highest circular polarization conversion efficiency, the optimization of the structural unit was conducted as follows. The parameters optimized included length, width, and periodicity. The optimization objectives were to achieve a phase difference of π between the two axes, maximize the transmittance, and minimize the transmittance difference. The Particle Swarm Optimization (PSO) algorithm was employed to determine the optimal values. The optimization process is illustrated in Fig. S2.

Considering this, individual unit optimization is conducted by employing periodic boundary conditions while also accounting for the efficiency impact induced by rotational effects. Based on this consideration, supercell optimization is performed, which, although computationally intensive, more accurately reflects the practical outcomes. Ultimately, the procedure entails initially optimizing the individual structural unit, then fixing the period and identifying the structural unit with the lowest zeroth-level occupancy ratio.

# Supplementary Note 3: Fabrication process


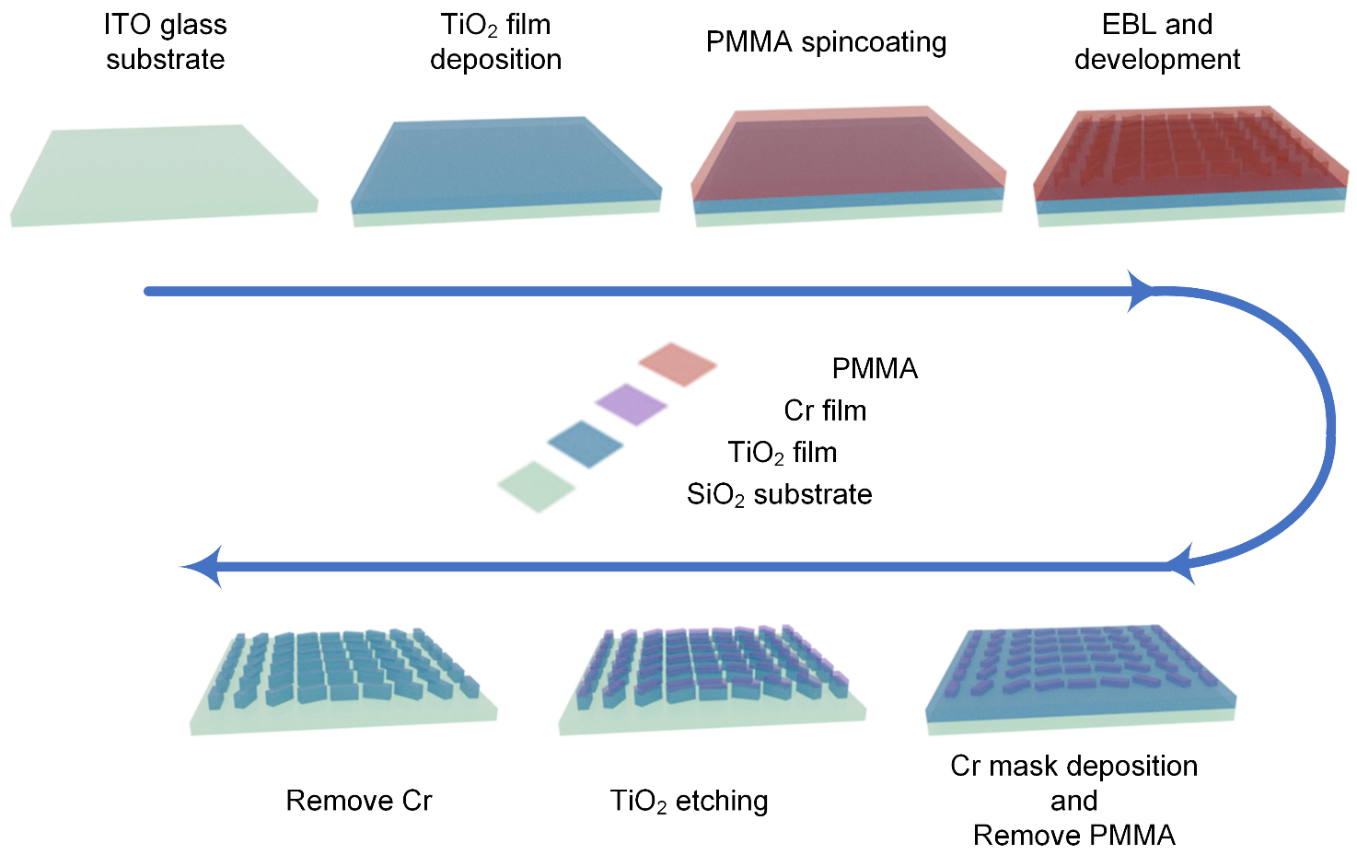


**Fig. S3:** The fabrication process of PB phase metasurface

The detailed fabrication process of the TiO_2_ metasurface is outlined in Fig. S3. Initially, a 960 nm TiO_2_ film is deposited onto an ITO glass substrate at a deposition rate of 0.6 Å/s. Subsequently, an 80 nm PMMA film is spin-coated onto the substrate and baked at 180 °C for one hour. The PMMA resist is then exposed to an electron beam (Raith E-line, 30 kV) and developed in a MIBK/IPA solution at 0 °C for 30 seconds to create the desired nanostructures. Next, a 30 nm Cr film is applied as a hard mask using an electron beam evaporator at a deposition rate of 0.3 Å/s, followed by a lift-off process in the PG remover solution. After the lift-off of the mask, reactive ion etching (Oxford 800 Plus) is employed to etch the TiO_2_ layers. In the final step, the residual Cr film is removed by immersing the sample in a chromium etchant for 10 minutes.

# Supplementary Note 4: Selecting the appropriate type of crystal and measurement basis

The process of generating entangled two-photon states can be simply described as a two-photon state under circular polarization basis. This is because the |*HV* ⟩ photon state can be directly written in the circular polarization basis as shown in equation S1:

$\left| HV \right\rangle=\frac{1}{2}\left( e^{-i2\theta}\left| RR \right\rangle-\left| LR \right\rangle+\left| RL \right\rangle-e^{i2\theta}\left| LL \right\rangle\right)\to\frac{1}{2}\left( e^{-i2\theta}\left| RR \right\rangle-e^{i2\theta}\left| LL \right\rangle\right)$ （*S1*）

The detailed calculation is depicted in the Fig. S4.


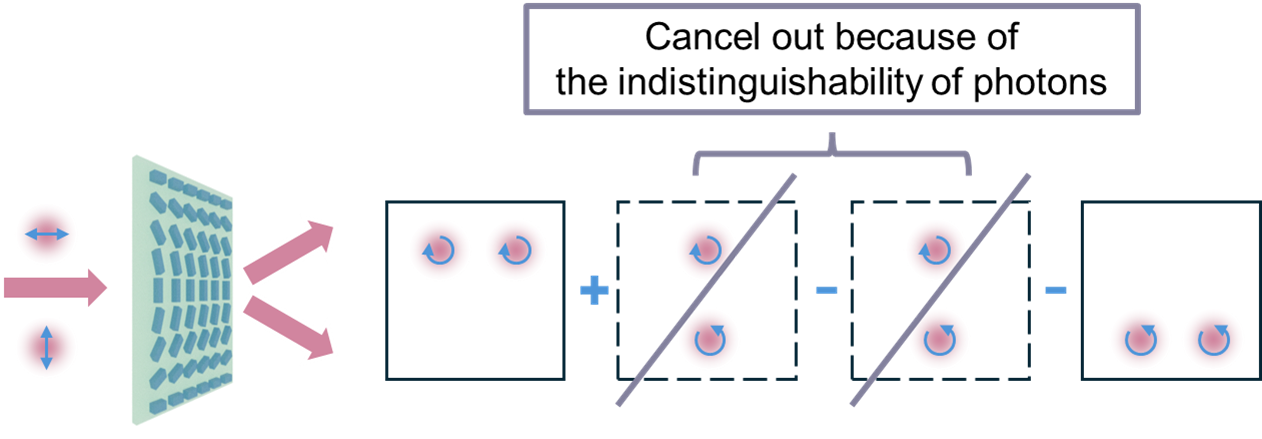


**Fig. S4.** Working principle of HOM interference in the polarization gradient Metasurface

We employ a type-II periodically poled potassium titanyl phosphate (PPKTP) crystal to achieve parametric down-conversion (PDC). This process generates two orthogonally polarized photons $\left| HV \right\rangle$, on the PB phase metasurface, the photon pairs experience HOM interference, and thus generate a two-photon state in circularly polarized basis as $\frac{i}{2}(\left| RR \right\rangle-e^{i\varphi}\left| LL \right\rangle)$, where $\varphi$ is a global phase between two-photon states. To accurately analyze these generated photons, it is essential to use appropriate measurement bases. The selection of suitable measurement bases allows for two times the frequency than the classical case.

Assuming the incident light consists of two components, $\left| H \right\rangle$ and $\left| V \right\rangle$, we express the incident light in the left circularly polarized (LCP, $\left| L \right\rangle$) and right circularly polarized (RCP, $\left| R \right\rangle$) basis.

$$\begin{aligned} \left| H \right\rangle=\frac{1}{\sqrt{2}}\left( \left| R \right\rangle+\left| L \right\rangle\right), \left| V \right\rangle=\frac{i}{\sqrt{2}}\left( \left| R \right\rangle-\left| L \right\rangle\right)\#\left( S2 \right) \end{aligned}$$

Upon passing through a metasurface, the photons undergo polarization conversion and acquire an additional phase due to the geometric phase, where θ is the rotation angle of the meta unit. The meta unit works as a half-wave plate (HWP).

$$\begin{aligned} \left| H^{'} \right\rangle=\frac{1}{\sqrt{2}}\left( e^{-i2\theta}\left| L \right\rangle+e^{i2\theta}\left| R \right\rangle\right), \left| V^{'} \right\rangle=\frac{i}{\sqrt{2}}\left( e^{-i2\theta}\left| L \right\rangle-e^{i2\theta}\left| R \right\rangle\right)\#\left( S3 \right) \end{aligned}$$

Subsequently, the light traverses an optical path that includes passing through two quarter-wave plates (QWP) and another metasurface. The objective is to ensure that the returning light exhibits opposite helicity compared to the incident light.

$$\begin{aligned} \left| H^{''} \right\rangle=\frac{1}{\sqrt{2}}\left( e^{-i4\theta}\left| L \right\rangle+e^{i4\theta}\left| R \right\rangle\right), \left| V^{''} \right\rangle=\frac{i}{\sqrt{2}}\left( e^{-i4\theta}\left| L \right\rangle-e^{i4\theta}\left| R \right\rangle\right)\#\left( S4 \right) \end{aligned}$$

Finally, the returning photons are projected onto the $\left| H \right\rangle$ and $\left| V \right\rangle$ basis for analysis.

$$\langle H\left| H^{''} \right\rangle=\frac{1}{2}(e^{-i4\theta}+e^{i4\theta})=\cos4\theta$$

$$\left\langle V | H^{''} \right\rangle=-\frac{i}{2}\left( -e^{-i4\theta}+e^{i4\theta} \right)=\sin4\theta$$

$$\langle H\left| V^{''} \right\rangle=\frac{i}{2}(e^{-i4\theta}-e^{i4\theta})=\sin4\theta$$

$$\begin{aligned} \left\langle V | V^{''} \right\rangle=-\frac{1}{2}\left( e^{-i4\theta}+e^{i4\theta} \right)=-\cos4\theta\#\left( S5 \right) \end{aligned}$$

In our case, we use type-II PPKTP and $\left| HV \right\rangle$ state, measured by $\left\langle HH| \right.$, the measured coincidence intensity is shown as

$$\begin{aligned} C_{\left\langle HH \mid H''\text{V}'' \right\rangle}=\left| \left\langle HH \mid H''\text{V}'' \right\rangle\right|^{2}=\left| \cos4\theta\sin4\theta\right|^{2}=\frac{1}{8}\left( 1-\cos16\theta\right)\#\left( S6 \right) \end{aligned}$$

As a comparison, if we use type-0 PPKTP and $\left| HH \right\rangle$ state, measured by $\left\langle HH| \right.$, the measured coincidence intensity exhibits a component at double the frequency. However, this component contributes merely one-eighth to the total signal. The result is shown as

$$\begin{aligned} C_{\left\langle HH \mid H''H'' \right\rangle}=\left| \left\langle HH \mid H''H'' \right\rangle\right|^{2}=\left| \cos4\theta\cos4\theta\right|^{2}=\frac{1}{8}\left( 3+4 \cos8\theta+\cos16\theta\right)\#\left( S7 \right) \end{aligned}$$

The single channel measurement, as another example, has only single frequence signal,

$$\begin{aligned} C_{\left\langle H \mid H^{''} \right\rangle}=\left| \left\langle H \mid H^{''} \right\rangle\right|^{2}=\left| \cos4\theta\right|^{2}=\frac{1}{2}\left( 1+\cos8\theta\right)\#\left( S8 \right) \end{aligned}$$

All results show in Fig. S5.

**Fig. S5:** Measurement results in comparison among different types of crystal and measurement basis

# Supplementary Note 5: Efficiency and divergence angle of the sample

The function of the sample is essentially a polarization grating based on left and right circular polarization. When horizontally polarized light is incident, it is equally divided into left and right circularly polarized beams. This serves as the basis for testing the efficiency of the sample, aiming for optimal performance in quantum experimental setups that demand high efficiency.

Fig. S6 (a-c) show the optical microscope images of our sample and the corresponding far-field spots. The three light spots represent left-circularly polarized light, unmodulated horizontally polarized light, and right-circularly polarized light, respectively. The corresponding test optical setup is shown in Fig. S6(d). A scatterer was used to calibrate the edge of the objective at the back focal plane to test the divergence angle, which is shown in Fig. S6(e).

The measured efficiencies are presented in Table. S1. Transmission efficiency refers to the ratio of the energy of all light spots in each figure to the energy of the light spot without the sample. Conversion efficiency refers to the proportion of transmitted light that can be utilized, specifically the proportion of right-handed circularly polarized light. Overall efficiency is the product of these two efficiencies, representing the ratio of usable light to incident light. It can be observed that the divergence angles of samples with different periods vary significantly, but the overall efficiency remains consistent. To better demonstrate the effect of quantum super-resolution, we used samples with smaller periods in the formal experiment.


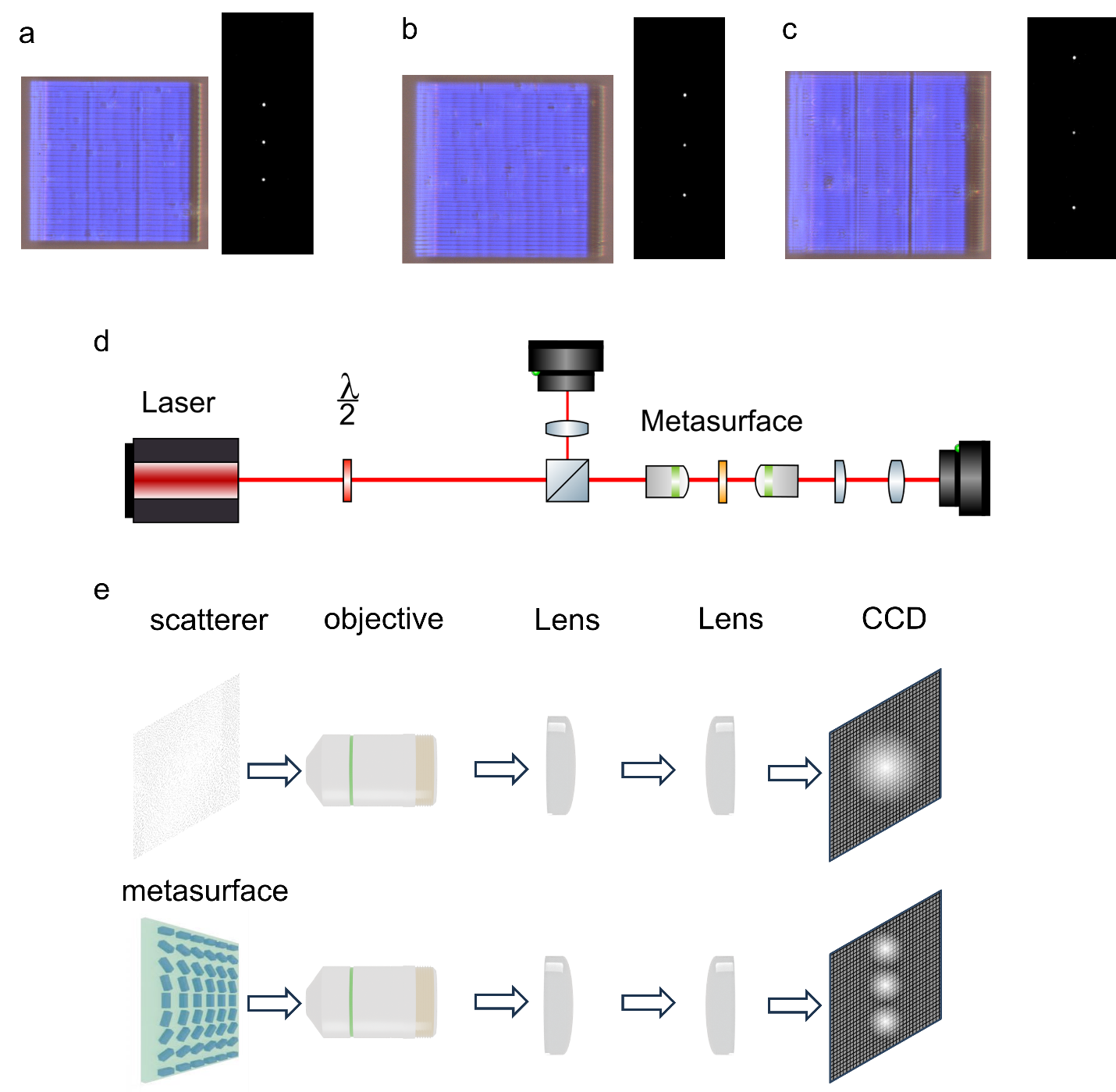


**Fig. S6:** Measurement results for samples with different periods and the optical setup. (a-c) Optical microscope photos and far-field light spots of samples with different periods. (d) Optical setup of testing efficiency and dispersion angle. (e) scatterer for testing the divergence angle

| Serial number | 1 | 2 | 3 |
| --- | --- | --- | --- |
| Divergence angle (deg) | 4.7067 | 6.1997 | 9.2987 |
| Transmission efficiency | 69.72% | 60.22% | 61.02% |
| Conversion efficiency | 68.11% | 76.33% | 78.45% |
| Overall efficiency | 47.49% | 45.96% | 47.87% |

**Table. S1:** Divergence angle and efficiency for the samples

# Supplementary Note 6: Two-photon behavior in experiments

As a verification experimental setup, we verify our experimental results by combining a two-photon source and a metasurface. First, a 405nm continuous laser is incident on a type-2 PPKTP crystal, generating a vertically polarized photon pair without time delay, which is incident on the metasurface through a lens group. The light after the metasurface generates a two-photon state, and then after reflection, the two beams of light interfere again on the metasurface, and the return photons are split, and then the coincident measurement is performed.


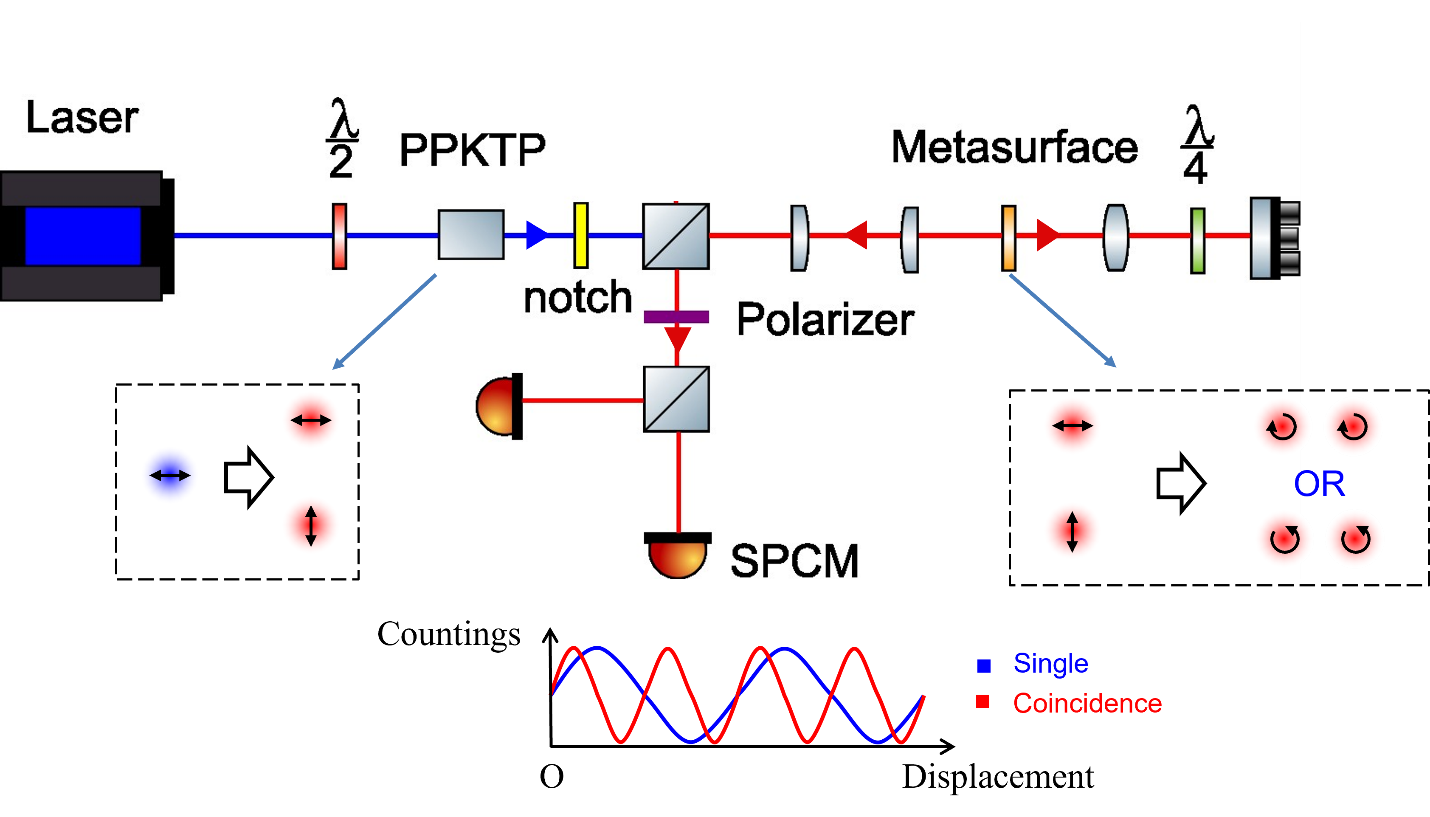


**Fig. S7:** The behavior of the two photons during the measurement

# Supplementary Note 7: Detailed interference scheme of the wavefronts

For the interference of wavefronts, the two beams have a tilted wavefront; that is, every point on the interference plane has a different, continuously changing phase. At the same time, the two beams are left-handed and right-handed circularly polarized, respectively, so we will get a rotating electric field vector on the interference plane. At this time, if the two are completely matched, the electric field will cancel each other, so we will get zero in the horizontal polarization direction. We will get a horizontal electric field component if the two are superimposed without being completely matched. Here, we assume that both wavefronts are periodic, so the interference effect we get should be sinusoidally related to the degree of mismatch.


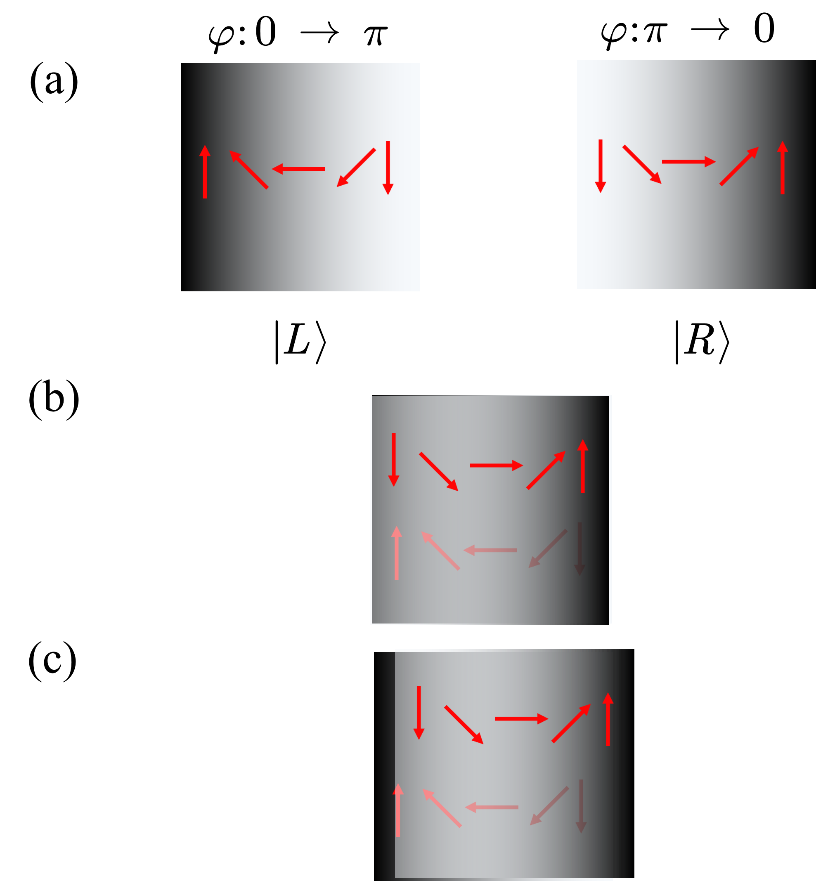


**Fig. S8:** Profile of the separated wavefronts. (a) The wavefronts are both inclined and inversely circularly polarized due to the effect of PB phase gratings. (b) Matched wavefronts. (c) Wavefronts with a small mismatch

# Supplementary Note 8: Error analysis

1. The error comes from the positioning error of the translation stage

We use a piezoelectric ceramic translation stage as a higher-level precision standard to measure the experimental accuracy of our system. However, the translation stage itself has positioning errors, which can also lead to inaccurate measurement results. The parameters of the translation stage are shown in Table. S2.

| Model | Piezoconcept LF1. 100 |
| --- | --- |
| Range of motion(μm) | 100 |
| Resolution(nm) | 0.1 |
| Noise floor(nm-typ.) | 0.01 |
| Repeatability(nm) | 0.2 |
| Linearization(typ.) | 0.02% |
| Resonant frequency(Hz) | 500 |
| Stiffness(N/μm) | 0.6 |

**Table. S2.** Parameters of the piezoelectric stage

The noise floor, linearization, and stiffness play significant roles in ensuring measurement precision. Additionally, our measurement process utilizes a step-by-step approach, operating at frequencies substantially lower than the resonance frequency of the Athens translation stage. Consequently, it is essential to set the drive unit of the translation stage to operate at a very low speed to prevent excessive vibrations, which could result in noise levels exceeding the standard values.

1. The error comes from the optical setup alignment

In this experiment, the process of spontaneous parametric down-conversion (SPDC) in a PPKTP crystal is employed to generate pairs of photons. Consequently, the optical path becomes considerably more complex and longer than that in corresponding classical light experiments. Additionally, to achieve interference results, it is necessary for the two photons to be projected into the same spatial mode, which restricts the use of single-mode optical fibers. As a result, the coupling efficiency of the optical path is subject to variation over time, contributing to measurement errors.

1. The error comes from the temperature fluctuation of the PPKTP crystal

The temperature of the PPKTP crystal has a direct impact on its two-photon yield. Our test results indicate that the two-photon yield at 20 degrees Celsius is 25% higher than at 23 degrees. To maintain the crystal's temperature close to 20 degrees, we utilize an oven to cool the base. However, due to the significantly higher ambient temperature, the crystal's temperature tends to fluctuate within a narrow range. As previously noted, variations in the two-photon yield have a direct effect on the accuracy of the measurement results.

1. The error comes from the inclination of the sample

Our PB phase grating features a periodic structure in a single direction; however, the alignment of the optical path does not guarantee that the phase gradient direction along the x-axis of the translation stage is consistent with that of the geometric phase grating. Consequently, any tilt of the sample may lead to a discrepancy between the actual grating period and the designed period, which could influence measurement accuracy.

# Supplementary Note 9: Modern photolithography alignment technology

| **Feature** | **Pre-Alignment** | **Alignment** | | **Overlay** |
| --- | --- | --- | --- | --- |
| Essence | Rough positioning | | Precise positioning/  calibration | Measurement/  evaluation |
| Purpose | Coarse wafer stage positioning | | Align reticle to reference marks | Measure inter-layer pattern deviation |
| Time | Before wafer fine movement | | Before exposure | After exposure/etch |
| Measurement Accuracy (typical value) | 6.5 μm  [*Fu, Z., et al. “A new wafer prealigner.” Robotic Welding, Intelligence and Automation.* Springer Berlin Heidelberg. 501-508(2007).] | | 10 nm  [*Optics Express*  28(5), 6755-6765(2020).] | Sub-nanometer level  [*Nanotechnology,* 36(20), 205301(2025)*.*] |
| Measurement Range (typical value) | Millimeter-level, wafer size | | 200 μm  [*Micromachines*  10(5), 339(2019).] | 10 nm  [*Measurement Science and Technology* 35(9), 095201 (2024).] |

**Table. S3.** Modern photolithography alignment technology

# Supplementary Note 10: Effect of changing the working wavelength of the metasurface

According to the equation $\Delta\varphi=\frac{2\pi}{\Lambda}\Delta x$, a shorter wavelength indeed corresponds to smaller meta-atoms. In theory, this could allow for a reduction in the period of the PB phase metasurface, potentially enhancing resolution, as shown in Fig. S9.

However, in the experiment, there are limitations to how much we can reduce the metasurface period. As the unit structures shrink to extremely small sizes, significant challenges arise concerning the fabrication precision required for electron beam lithography. In our method, we achieved an effective period of 2.5 μm, while the actual metasurface period is 5 μm. This was accomplished by amplifying the measured phase through two-photon state coincidence measurement, thereby enhancing the measurement resolution.


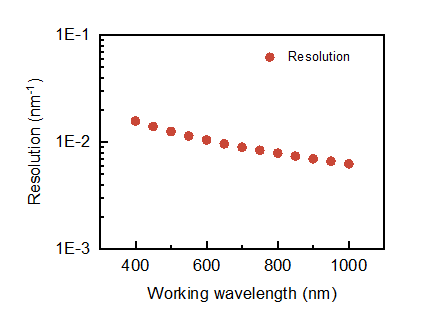


**Fig. S9.** Effect of changing the wavelength

# Supplementary Note 11: Working principle of coincidence counting

In our work, we perform the coincidence counting method on a two-photon state. By projecting the entangled two-photon states onto the horizontal polarization state as the working principle in the manuscript, we can extract the phase between different polarization states through coincidence measurement. The difference between two-photon absorption and coincidence counting is shown in Fig. S10.


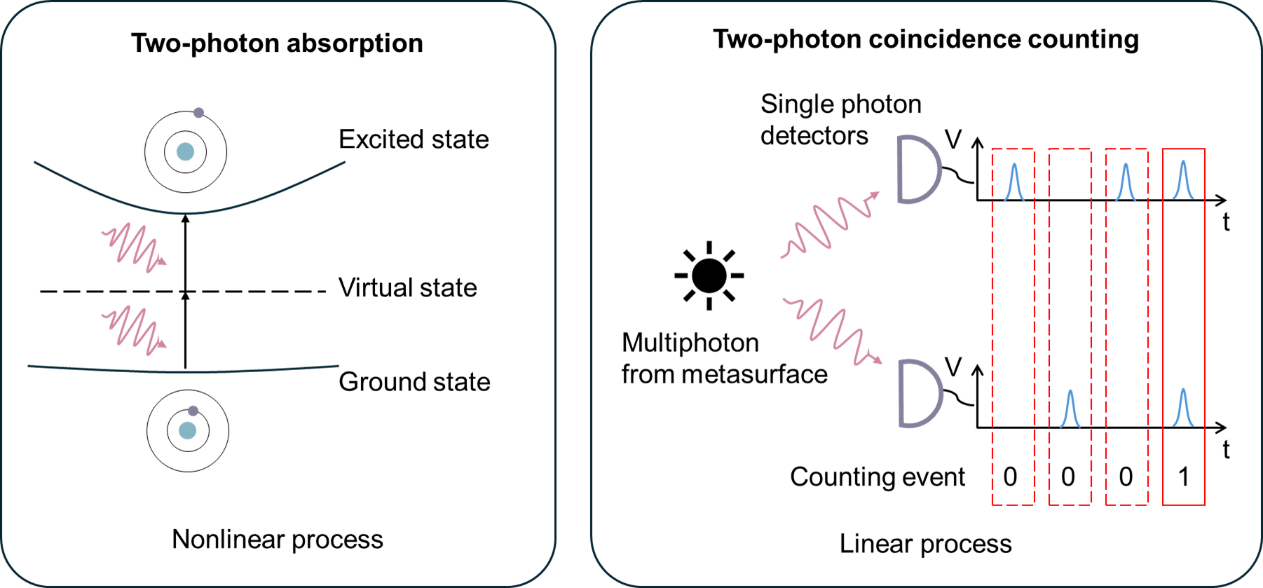


**Fig. S10.** The difference between two-photon absorption and coincidence counting
